# Age‐related ultrastructural changes of the basement membrane in the mouse blood‐brain barrier

**DOI:** 10.1111/jcmm.13980

**Published:** 2018-11-19

**Authors:** Laura Cristina Ceafalan, Tudor Emanuel Fertig, Teodora Cristina Gheorghe, Mihail Eugen Hinescu, Bogdan Ovidiu Popescu, Jens Pahnke, Mihaela Gherghiceanu

**Affiliations:** ^1^ Ultrastructural Pathology Laboratory ‘Victor Babeș’ National Institute of Pathology Bucharest Romania; ^2^ Department of Cellular, Molecular Biology and Histology ‘Carol Davila’ University of Medicine and Pharmacy Bucharest Romania; ^3^ Department of Neurology ‘Carol Davila’ University of Medicine and Pharmacy Bucharest Romania; ^4^ Department of Neuro‐/Pathology University of Oslo and Oslo University Hospital Oslo Norway; ^5^ LIED University of Lübeck Lubeck Germany; ^6^ Department of Bioorganic Chemistry Leibniz Institute for Plant Biochemistry Halle Germany; ^7^ Department of Pharmacology University of Latvia Medical Faculty Riga Latvia

**Keywords:** ageing, basement membrane, blood‐brain barrier, electron tomography

## Abstract

The blood‐brain barrier (BBB) is essential for a functional neurovascular unit. Most studies focused on the cells forming the BBB, but very few studied the basement membrane (BM) of brain capillaries in ageing. We used transmission electron microscopy and electron tomography to investigate the BM of the BBB in ageing C57BL/6J mice. The thickness of the BM of the BBB from 24‐month‐old mice was double as compared with that of 6‐month‐old mice (107 nm vs 56 nm). The aged BBB showed lipid droplets gathering within the BM which further increased its thickness (up to 572 nm) and altered its structure. The lipids appeared to accumulate toward the glial side of the BM. Electron tomography showed that the lipid‐rich BM regions are located in small pockets formed by the end‐feet of astrocytes. These findings suggest an imbalance of the lipid metabolism and that may precede the structural alteration of the BM. These alterations may favour the accretion of abnormal proteins that lead to neurodegeneration in ageing. These findings warrant further investigation of the BM of brain capillaries and of adjoining cells as potential targets for future therapies.

## 1 INTRODUCTION

The blood‐brain barrier (BBB) controls the molecular composition of the neuronal environment. BBB is composed of cellular elements, all in close contact with the interposing basement membrane (BM).

In the BBB, the endothelial cell BM encloses the pericytes and tightly attaches the astrocyte foot processes by specific adhesion molecules.^1^ The end‐feet of astrocytes overlap and cover almost the entire surface of the BM, forming an envelope around the capillaries. However, small gaps have been described on the surface of pericytes where the neuropil can directly contact the BM, as well as on the surface of endothelial tubes, where perivascular cell processes (most probably microglial processes) intermingle with the end‐feet of astrocytes and come into contact with the BM.^2^ Altogether, these cells form the neurovascular unit (NVU).^3^

The BM is a mandatory component of any tissue barrier by providing a filtering meshwork for diffusing molecules but also a side‐specific structural support for cell adhesion. Moreover, the BM interacts with tissue‐specific cell surface receptors mediating signals within or between different compartments, thereby regulating cell behaviour and survival.^4^ BM composition and structure is tissue‐specific and dynamic^5^ and undergoes compositional and structural changes with ageing.^6^

In the BBB, the BM comprises a complex network of extracellular matrix (ECM) molecules such as type IV collagen, laminins, fibronectin, heparan sulphate and proteoglycans.^1^, ^7^, ^8^

BM facilitates the intercellular interactions in a highly dynamic environment and undergoes transformations throughout animal life 9 and it is particularly important in transport across the BBB. An increasing body of evidence derived from both human and animal samples suggests that age‐associated cognitive decline in the absence of comorbidities is a consequence of specific changes in both composition and structure of the BBB, which may disrupt the barrier function and compromise molecular trafficking.^10^, ^11^ These changes may affect any component of the barrier, and are either a result of metabolic dysfunction or vascular or glial pathology which in turn may lead to alterations of the BM synthesis and degradation.^3^

Previous structural and ultrastructural studies reported various changes in endothelial cells that could affect the function of the BBB. Examples include tight junction loosening, suggested to be the primary reason for increased capillary permeability and subsequent influx of neuro‐inflammatory molecules,^11^ increased numbers of pinocytotic vesicles and decreased density of mitochondria, resulting in the accumulation of reactive oxygen species.^12^, ^13^

Ageing has also been reported to be associated with ultrastructural, molecular and biophysical changes of BM^14^ including the cerebrovascular BM. Alteration of the BM may also occur in various neurodegenerative diseases.^1^ Indeed, ageing BM becomes thicker and stiffer as the proportion of collagens and laminins changes.^14^ A recent study reported microvascular fibrosis, and amorphous fibrosis in the BM of the BBB in ageing.^8^ BM ultrastructural changes included focal thickening, splitting and duplication, as well as accumulation of amorphous material of unknown composition and of membranous inclusions identified as degenerated pericytes.^8^

This study focused on the ultrastructural changes of the BBB in ageing but otherwise healthy C57Bl/6J mice and revealed an unexpected accumulation of lipids in the BM of brain capillaries which, to our knowledge, was not previously reported.

## 2 MATERIALS AND METHODS

### 2.1 Animal model

The ultrastructural analysis was performed on cerebral tissue harvested from three different animals from each of two age groups: 6‐month and 24‐month‐old C57BL/6J mice (The Jackson Laboratory, Bar Harbor, ME, USA, strain no. 000664). The study was approved by the ethics committee of the Victor Babeș National Institute of Pathology. All animals were declared healthy, with no associated comorbidities and on normal ad libitum diet and housing conditions. To minimize unnecessary suffering, the mice were killed by cervical dislocation. The brain was removed in less than 1 minute and further processed for electron microscopy and light microscopy.

### 2.2 Tissue sampling for electron microscopy

Thin slices (about 1 mm thick) from the mice brains were made on the coronal plane at the base of the posterior hypothalamus and frontal lobe (Bregma location: 1.00 mm). The slices were promptly immersed in 4% buffered glutaraldehyde (buffer 0.1 mol L^−1^ sodium cacodylate at pH 7.3) at room temperature (RT) and further sectioned in 1 mm thick strips. Two arbitrary strips from each hemisphere were further grated in 1 mm^3^ fragments. The fixation by immersion was preferred to fixation by perfusion to avoid structural artefacts generated by the sudden distension of capillaries because of hypervolaemia (endothelial cell flattening, basement membrane distension, flattening of any virtual drainage space around capillaries).

### 2.3 Transmission electron microscopy (TEM)

The 1 mm^3^ fragments of brain tissue were fixed by immersion in 4% glutaraldehyde, buffered with 0.1 mol L^−1^ sodium cacodylate (pH 7.3) at 4°C overnight and 25 pieces were further processed for Epon‐embedding (Agar100 resin, Agar Scientific, Essex, UK) as previously described.^15^ Epon‐embedded murine cerebral tissue fragments were sectioned using a Leica EM UC7 ultramicrotome (Leica Microsystems GmbH, Vienna, Austria). Light microscopy was done on 1‐μm‐thick sections stained with 1% toluidine blue and representative images were recorded under a Nikon 600 light microscope performed with a 100× objective (oil immersion) with a 11‐megapixel camera (AxioCam HRc, Zeiss, Germany). Random areas from the brain tissue of both 6‐month and 24‐month‐old mice were oriented for ultrastructural analysis. Five Epon‐embedded blocks from each mouse were sectioned for TEM and mounted on 50‐mesh copper grids (Agar Scientific). Electron microscopy imaging was performed on 60 nm ultra‐thin sections counterstained with uranyl acetate and Reynolds lead citrate (Agar Scientific) at 80 kV on a Morgagni 268 TEM (FEI Company, Eindhoven, The Netherlands), equipped with a MegaView III CCD (Olympus, Germany) and running iTEM‐SIS software (Soft Imaging Systems, Olympus, Germany).

### 2.4 Assessment of basement membrane (BM)

A cortical capillary was defined as a vascular structure with an inner diameter lower than 7 μm, with the lumen bordered by endothelial cells, delimited by a BM and enclosed pericytes (cell body and/or processes). A clear distinction between capillaries and small post‐capillary venules was challenging in TEM, as both vessel types are surrounded by pericytes. A distinction was made based on their dimensions; capillaries were considered to have a lumen below 5‐7 μm and post‐capillary venules a diameter above 7 μm. Transversally sectioned capillaries were imaged at 22,000 × −44,000× nominal magnification. Image analysis and assessment of BM thickness (Supplementary files Figure S1) were performed with AnalySIS software (Soft Imaging Systems). The BM membrane between astrocytic end‐feet and endothelial cells or pericytes was measured at the points where the cellular membranes were clearly visible (about 3 measurements per capillary). Oblique sectioned BMs were avoided, as at this level the cellular membranes of endothelial cells and astrocytes are blurred. About 100 BM thickness measurements were done for each of the two mouse age groups and results were expressed as mean ± SD (Supplementary files Figure S2). The datasets were compared using a two‐tailed *Z*‐test (significance set at *P* < 0.05).

### 2.5 Super‐resolution microscopy

BODIPY 493/503 (D3922, Invitrogen, Carlsbad, CA, USA) was used to verify the lipid nature of the nm‐sized droplets in the BM of brain capillaries, as it is rapidly partitioned in the non‐polar environment of lipid droplets. Brain hemispheres were embedded in Surgipath Cryo‐Gel (Leica Microsystems) and frozen at −27°C in a cryostat (CM 1510S, Leica Mycrosystems). Five micrometre thick sagittal sections of aged mouse brains were fixed with 4% paraformaldehyde after sectioning for 20 minutes and then incubated with 10 μg/mL BODIPY 493/503 in 150 mM NaCl for 1 hour at RT. After washing in PBS, sections were incubated with polyclonal rabbit anti laminin primary antibody (1:100, Sigma, L9393, Munich, Germany) for 2 hours at RT and then for another hour with AlexaFluor 532 conjugated goat anti‐rabbit antibody (1:400, Invitrogen, A11009). Sections were mounted with SlowFade™ Gold antifade mountant with DAPI (Invitrogen, S36939) and analysed by super‐resolution microscopy on the Leica TCS SP8 STED 3× module with 660 nm STED laser line (Leica Microsystems GmbH, Germany), in the sequential scan mode, performed with an oil immersion HC PL APO CS2 100×/1.40NA objective. A 405‐nm‐UV‐laser was used for imaging DAPI, whereas for Bodipy and AlexaFluor 532 optimal excitation wavelengths were selected using a white light laser (tunable range between 470 and 670 nm, at 1 nm intervals). Emission was registered using HyD detectors. Image acquisition and 3‐D rendering of image stacks were done using the manufacturer supplied LASX software (Leica Microsystems GmbH) and deconvolution with Huygens package (Scientific Volume Imaging, Hilversum, the Netherlands).

### 2.6 Electron tomography and 3‐D modelling

For electron tomography, 300 nm sections were mounted on 75‐mesh carbon coated grids. Imaging was performed using a 200 kV Talos F200C TEM (FEI Company), equipped with a 4K × 4K Ceta camera and using manufacturer supplied tomography software (FEI Company). The unbinned pixel size at specimen level was 0.66 nm, at 22,000× nominal magnification. Single axis tilt series were recorded between −55° and +55°, at 1° intervals. Tilt series images were aligned and reconstructed in IMOD‐eTOMO, using the Back Projection algorithm.^16^ Prior to imaging, 15 nm colloidal gold particles (Aurion, Wageningen, the Netherlands) were added on both sides of each section to assist alignment. Contours of subcellular structures in one tomogram were manually traced on 210 virtual slices, to obtain a 3‐D model. Measurements were done using *imodinfo*. The final movie was generated using the free version of the software VideoMach (http://gromada.com/videomach/).

## 3 RESULTS

### 3.1 Light microscopy

**Figure 1:**
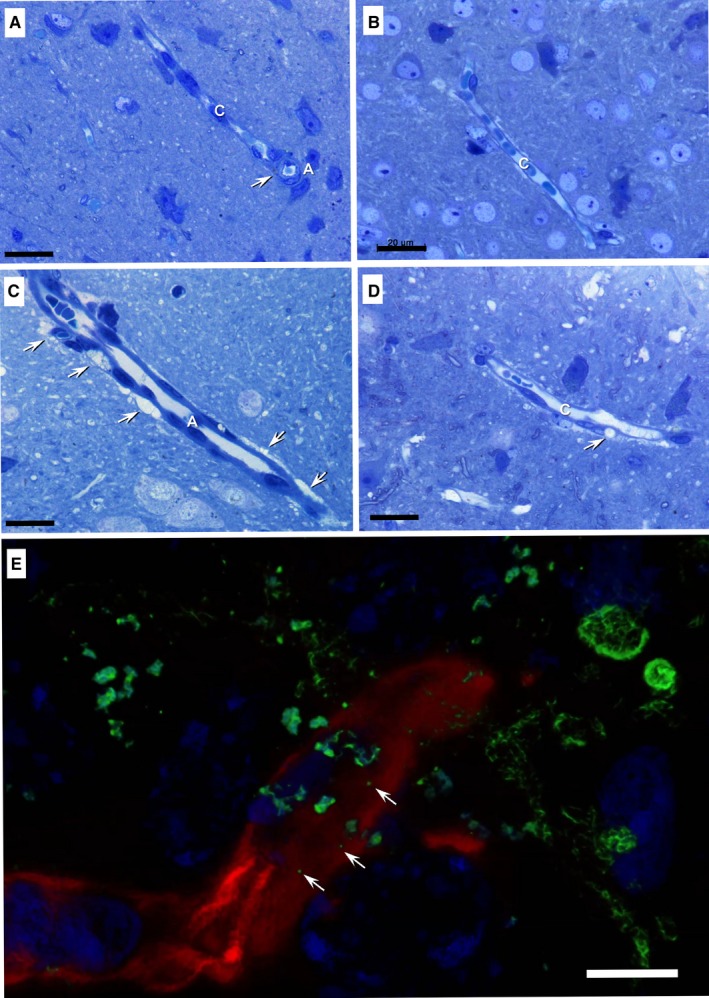
Light microscopy on toluidine‐blue‐stained semithin sections of plastic embedded brain tissue from 6‐month‐old mice (A, B) and 24‐month‐old mice (C, D, E). Rare lipid droplets were spotted in the vascular smooth muscle cells of arterioles in the young mouse brain samples (arrow in A). Lipid accumulations (arrows) are visible in smooth muscle cells of arterioles (C), and pericytes (D). A—arteriole, C—capillaries. (E) 3‐D rendered image stack of brain capillaries from a 24‐month‐old mouse brain. Double immunofluorescent labelling for lipid droplets (Bodipy 493/503, green) and laminin from the basement membrane (AlexaFluor 532, red) shows nanometer‐sized lipid droplets (arrows) immersed in the basement membrane. Nuclei were stained with DAPI (blue). Scale bars: 20 μm; E—5 μm

The brain tissue was first examined by light microscopy on 1 μm semithin plastic sections stained with toluidine blue (Figure 1). No significant vascular changes were observed in the brain tissue from 6‐month‐old mice (Figure 1A, B). Small and rare lipid droplets were visible in vascular smooth muscle cells (Figure 1A). Discrete swelling of the end‐feet of astrocytes surrounding capillaries was also visible (Figure 1B), likely as a result of tissue fixation by immersion instead of perfusion.

Light microscopy on brain tissue from 24‐month‐old mice showed frequent vascular smooth muscle cells, located in the wall of arteries and arterioles, loaded with lipid droplets (Figure 1C, D). Lipid droplets were also present in pericytes (Figure 1D).

### 3.2 Super‐resolution STED microscopy

The lipid nature of various droplets accumulating in the brain of 24‐month‐old mice was assessed by Bodipy 493/503 fluorescent staining on cryosections. The BM was highlighted by fluorescent staining of laminin. Large droplets were detected in the cytoplasm of different cells from the cerebral cortex as well as in perivascular cells of both capillaries and arterioles. However, nm‐sized lipid structures and aggregates were also visualized in the thickness of the BM of small cortical capillaries (Figure 1E; Supplementary Figure S4 and Video S2).

### 3.3 Transmission electron microscopy (TEM)

**Figure 2:**
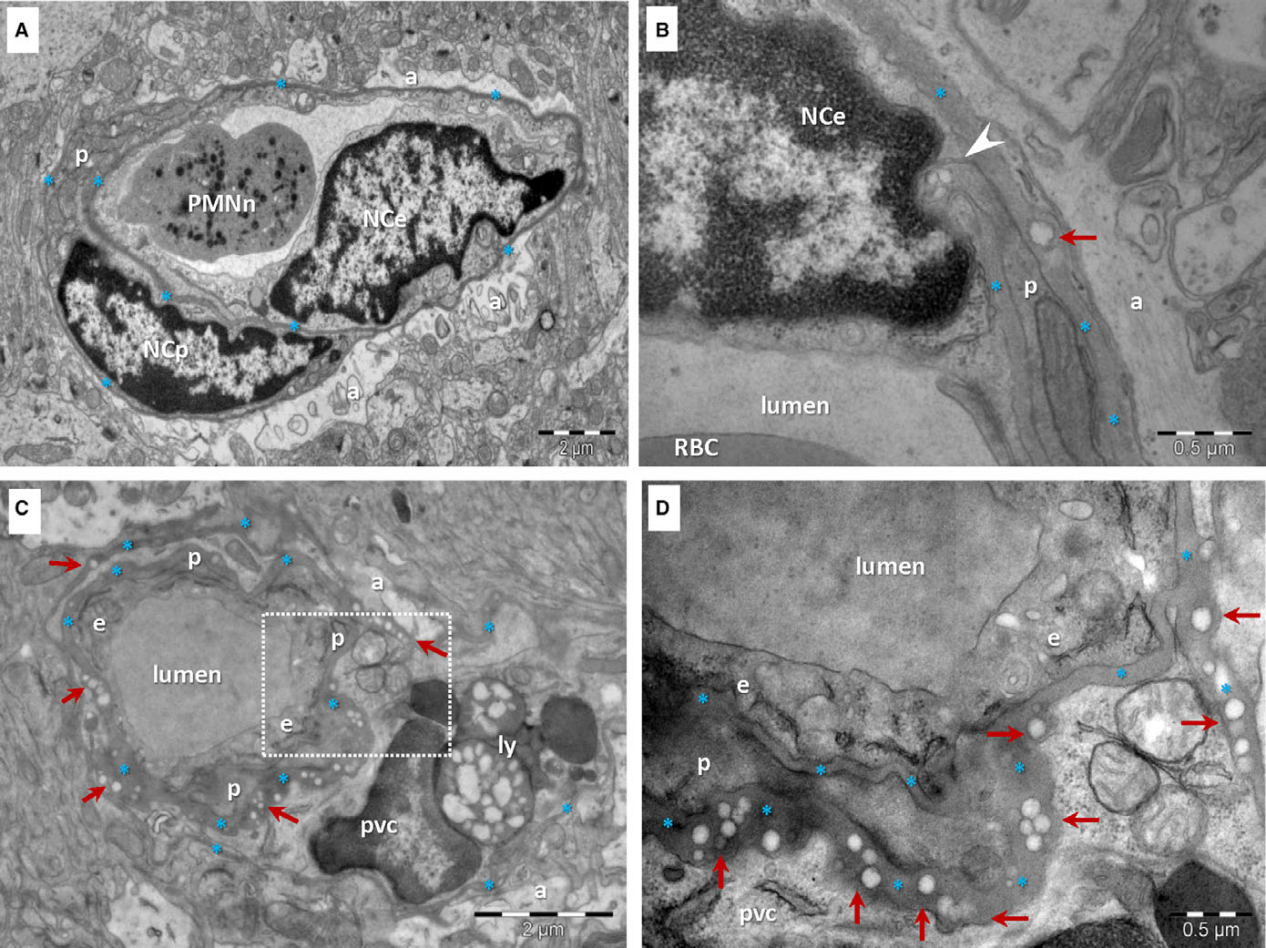
TEM of brain capillaries in a 6‐month‐old mouse (A, B) and a 24‐month‐old mouse (C, D). A, A capillary from the 6‐month‐old mouse has a uniform BM (*) subjacent to an endothelial cell (NCe—nucleus of the endothelial cell; e—endothelial cell) and enclosing a pericyte (p). PMNn—polymorphonuclear neutrophil; a—end‐feet of astrocytes. B, Rare droplets (arrow) may be seen in the BM (*) of capillaries from the young mouse brain. Arrowhead indicates a direct contact between a pericyte and an endothelial cell. C, The BM (*) of brain capillaries from the aged mouse is thicker, uneven and contains numerous electron‐lucent, single or grouped droplets (arrows). Large lipid‐containing lysosomes (ly) are present in a perivascular cell (pvc). p –pericyte; a—end‐foot of astrocyte. D, Higher magnification of marked area in C

The TEM ultrastructural analysis of brain samples from 6‐month‐old mice showed no notable changes in the appearance of large blood vessels and capillaries (Figure 2A) other than infrequent small lipid droplets (~100 nm in diameter) in the thickness of the BM (Figure 2B). The thickness of the BM was 56.78 ± 12.50 nm (min: 30.58 nm; max: 96.95 nm; n = 100).

In the 24‐month‐old mice brain tissue (10 different areas) the vascular smooth muscle cells of arterioles and the pericytes or perivascular cells (Figure 2C) were frequently loaded with lipid droplets. Perivascular cells were often observed embedded in the BM of capillaries (Figure 2C) in conjunction with pericytes. These perivascular cells with a clear cytoplasm presented numerous lipid‐containing lysosomes (lipofuscin). The brain capillaries had more irregular contours (Figure 2C) compared with those of younger mice (Figure 2A). The BM of capillaries proved to be thicker than in the brain of young mice (*P* < 0.001 two‐tailed *Z*‐test, Supplementary Figures S1 and S2) and appeared uneven because of accumulation of electron‐lucent inclusions (Supplementary files Figure S1). Moreover, the BM appeared to send small extensions between the end‐feet of astrocytes (Figure 2C, Supplementary files Figure S2).

The median thickness of aged BM was 107.53 ± 23.76 nm (min: 65.82 nm; max: 159.82 nm; n = 100) in the segments with no structural abnormalities. The thickness of the BM in the segments containing lipid droplets was even higher: 268.18 ± 118.29 nm (min: 150.90 nm; max: 572.07 nm; n = 100).

**Figure 3:**
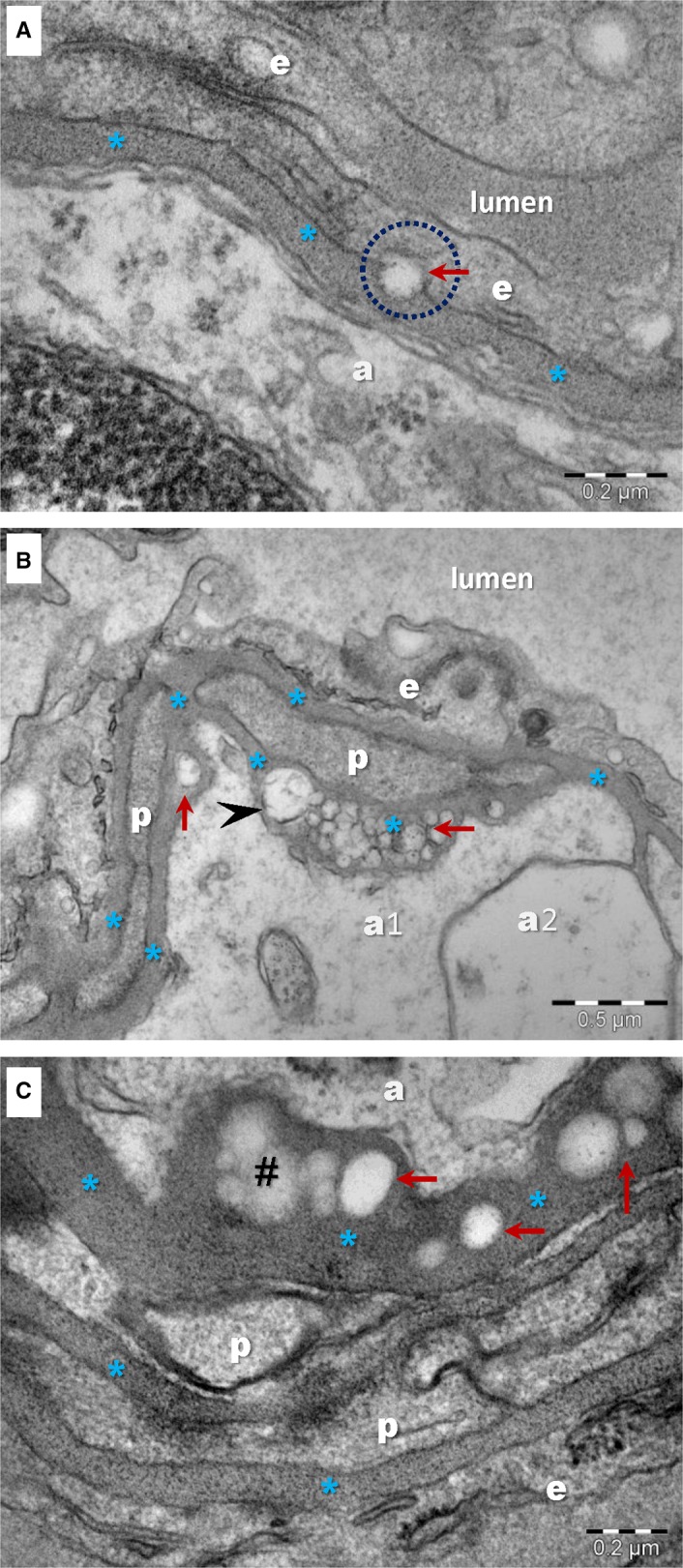
TEM images show single or grouped droplets (arrows) visible in the BM of brain capillaries in aged mice. Accumulation of droplets alters the ultrastructure of the BM (*) and increases the distance between endothelial cells (e) or pericytes (p) and the end‐feet of astrocytes (a). A, A droplet seems to be delivered by/to the endothelial cell (encircled). There is no clear membrane (bilayer) lining the droplet. B, There is variance in lipid droplet dimensions, whereas most range between 100 and 200 nm, rare larger droplets could be observed (arrowhead). C, Weak electron‐dense structures (#) with irregular and indefinite contours could be seen altering the ultrastructure of the BM (*)

Electron‐lucent droplets were visible in the thickness of the BM of about 25% of capillaries (19 out of 75) in aged brain samples (Figures 2C, D and 3). Such inclusions were not lined by a clear bilayered membrane but rather by a single layer of phospholipids (Figure 3A, C). Their electron‐lucent appearance is consistent with a lipid composition, as lipids are extracted during processing for plastic embedding of biological samples for electron microscopy. The dimension of the lipid droplets within the BM of BBB in aged mice was 97.32 ± 25.48 nm (min: 50.40 nm; max: 157.15 nm; n = 100). They were usually found as isolated droplets (Figure 3A) or small clusters (Figure 3C), but larger aggregates were also detected (Figure 3B).

Frequently, these lipid droplets were gathered toward the end‐feet of astrocytes either between endothelial cells and the end‐feet of astrocytes (Figures 2D and 3A) or between pericytes and the end‐feet of astrocytes (Figures 2D and 3B, C). The BM between pericytes and endothelial cells seemed to be free of lipid droplet accretion (Figures 2C and 3B; Figure S1). There was no clear connection between lipid droplets within the BM and nearby cellular membranes of endothelial cells, pericytes or end‐feet of astrocytes. One single image showed a lipid droplet in relation with an endothelial cell (Figure 3A).

In addition to the accumulation of lipid droplets, the infrequent deposition of a moderate electron‐dense, amorphous material was also found to contribute to the thickening of the BM (Figure 3C). The nature of these BM inclusions cannot be speculated based solely on TEM images.

### 3.4 Electron tomography (ET)

To investigate the spatial distribution of the lipid droplets within the BM and their relation with cells of the BBB (endothelial cells, pericytes and end‐feet of astrocytes) we performed ET (Supplementary files Figure S3).

**Figure 4:**
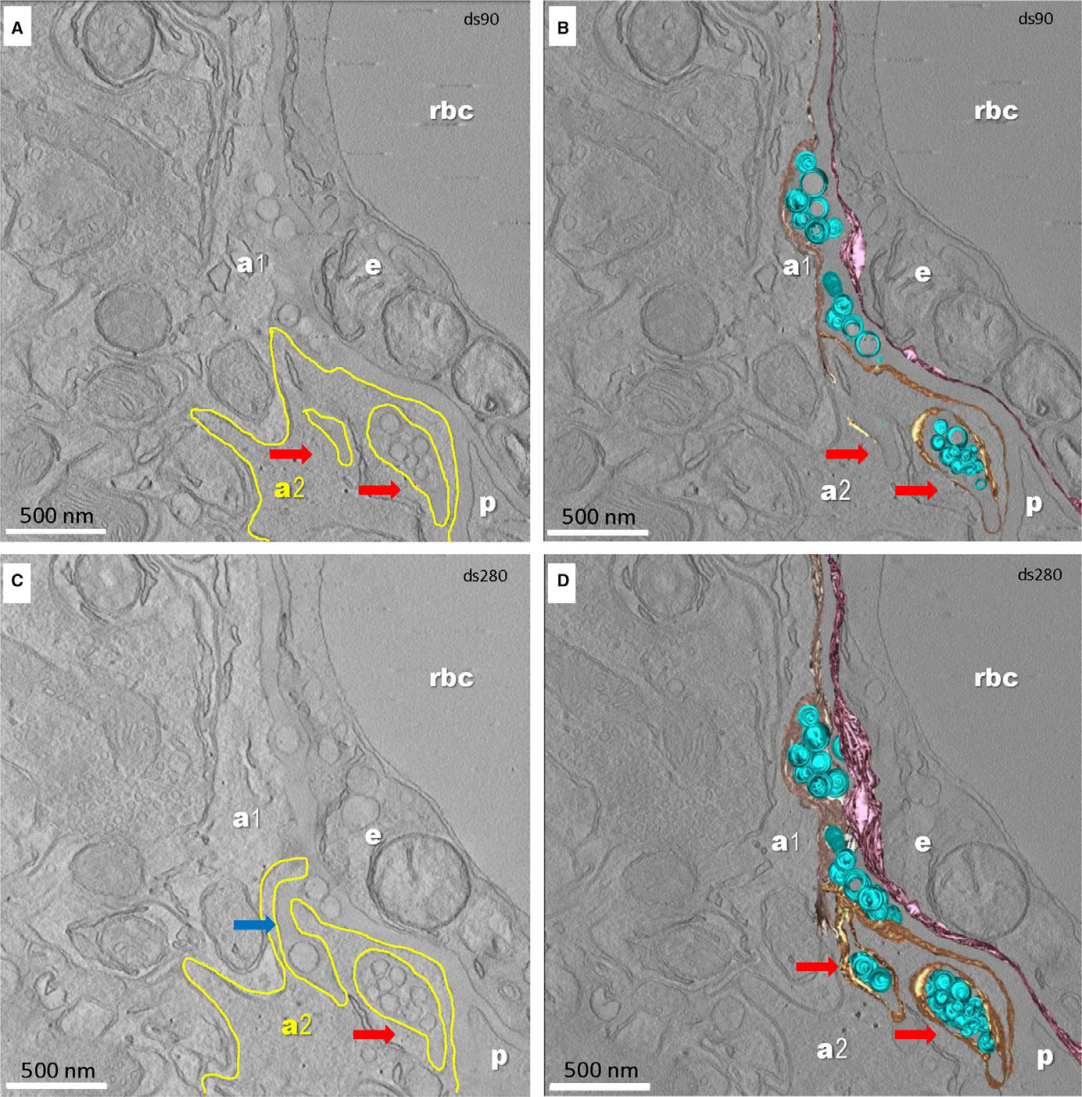
Sections from the ET of a segment of brain capillary (24‐month‐old mouse). Digital slice 90/300 (A, B) and 280/300 (C, D) from the tomographic volume (A, C) and 3‐D reconstruction (B, D). a1, a2—end‐feet of astrocytes; e‐endothelial cell; p—pericyte; rbc—red blood cell. The digital slices (A, B) show that the end‐foot of astrocyte a2 (plasma membrane outlined with yellow) folds around segments of the BM containing lipid droplets within recesses (arrows). One extension of the BM in a pocket of the end‐foot process of astrocyte a2 is clearly visible in C (blue arrow). The 3‐D reconstruction from a 300 nm thick section (D) shows numerous lipid droplets in the BM. Colour code: lipid droplets in the BM shown as blue spheres; the plasma membrane of the endothelial cell facing the BM shown in purple; plasma membrane of the end‐feet of astrocytes facing the BM is lined with beige for astrocyte a1 and yellow for astrocyte a2. Scale bar—500 nm

ET confirmed the uneven width of the BM of aged BBB and showed that lipid droplets form clusters in the thickness of the BM (Figure 4; Video S1). ET also showed no obvious contacts between any lipid droplet within the BM and nearby cellular membranes of endothelial cells, pericytes or end feet of astrocytes.

Notably, ET revealed that the end feet of astrocytes around aged brain capillaries form recesses that accommodate extensions of the BM containing aggregates of lipid droplets (Figure 4; Video S1).

## 4 DISCUSSION

Our ultrastructural study of the BBB in ageing mice model reveals the build‐up of lipid inclusions within the BM and its increased thickness.

The BM is synthetized by endothelial cells and pericytes, but astrocytes may be also involved in BM homeostasis.^17^ Pericyte–astrocyte interactions seem to regulate the integrity of the endothelial tight junction and BM.^18^ Some studies have found evidence that pericyte loss with ageing is associated with BBB breakdown in mice, but loss of pericyte coverage in the human brain capillaries was shown to have no correlation with age.^19^ We can speculate that the loss of pericytes may stimulate BM synthesis by endothelial cells leading to an increase in the BM thickness with age.

Similar changes have been previously described and analysed in a series of reports regarding Bruch's membrane in samples from elderly persons diagnosed with age‐related macular degeneration. Bruch's membrane is a more complex, layered ECM compartment functioning as a physical and semipermeable barrier between the retinal pigment epithelium (RPE) and the choroid containing capillaries that supply the RPE and the outer layers of the retina.^20^ In time, this membrane suffers a number of morphological changes, which favour RPE detachment and vascularization of the subretinal spaces. It has been reported that with age Bruch's membrane increases in thickness^21^ as structural proteins like elastin and collagen degenerate and neutral lipids start to accumulate. Lipid accumulation is more probably responsible for the subsequent RPE detachment^22^ by building up a hydrophobic barrier.^23^

There is no obvious evidence on the source of the accumulating lipid particles in BBB, whether they are produced locally or systemically. Is the thickening of the BM preventing the clearance of lipids by endothelial cells or could we infer a declining demand for fatty acids (FA) in the ageing brain? FA are not the first choice for energy production even in the normal young brain tissue.^24^ However, polyunsaturated FA functioning as essential signalling molecules are not synthesized in brain cells. Recent models suggest that FA crossing the BBB are not derived from circulating lipoproteins (LPs), as they are not taken up by brain cells, except for some of the smaller circulating HDL.^25^ Lipoproteins should be first hydrolysed within the endothelial cells of brain capillaries^26^ and then FA are transported across the endothelium with^27^ or without a protein transporter.^28^

The overall ultrastructural appearance of the particles entrapped in the aged BM of the BBB, characterized by a unilaminar coating and electron‐lucent content, is highly suggestive for a lipoprotein assembly. These lipid droplets might be a mechanism for lipids transfer through the BM of BBB. Moreover, the pattern of lipid particle build‐up only toward the glial side of the BM suggests a probable origin in the nervous tissue, rather than systemic delivery via capillaries.

A recent study^19^ demonstrated an increase in gliosis and GFAP expression associated with ageing in mice, but did not observe a change in the number or morphology of astrocytes. Astrocytes are the major source of lipoprotein constituents and lipoprotein assembly in the brain.^25^ ApoE and ApoJ/clusterin are primarily expressed in astrocytes in mice where the mRNA and protein expression of both LPs are affected by age by increasing the production of ApoJ and dropping ApoE levels,^29^ influencing the production of LPs as well as the clearance of Aβ.^30^

We also detected the presence of unusual membrane pockets made by the end‐feet of astrocytes containing projections of the adjacent BM and filled with large aggregates of lipid droplets, suggesting that astrocytes may be part of a clearance mechanism.

Whether the lipid accumulation represents the cause or the consequence of the local damage of the NVU remains to be determined. However, the accumulation of such lipid particles, as in the case of Bruch's membrane, may eventually generate a hydrophobic obstacle for the molecular traffic through the BBB, thereby changing the composition of the microenvironment for the NVU. On the long run, these changes further generate or amplify neuronal injury. Considering regional differences reported in the morphological and functional reactivity of brain capillaries^31^ future studies should focus on the specific areas involved in neurodegenerative diseases. Moreover, in the light of the function of the recently described glymphatic system^1^ as a specific compartment in the BM,^32^ accumulation of lipids within BBB might interfere with the capacity of the brain to remove altered proteins, resulting in progression of neurodegeneration.

The lipid composition of the BBB in aged mice as well as its potential correlation with a specific serum lipid profile remains to be determined by future studies, so too the translation of these observations in human subjects. An ultrastructural approach can only open new perspectives on the potential alterations leading to BBB dysfunction in older patients.

Ageing of BBB in mice involves substantial BM changes, the most striking of which is a doubling of its thickness. In some capillaries, BM thickness is further increased by the accumulation of lipids forming isolated droplets or large aggregates. These changes may reflect an imbalance in lipid metabolism and may precede and favour the accumulation of the altered proteins in the brain and subsequent neurodegeneration.

## CONFLICT OF INTEREST STATEMENT

The authors confirm that there are no conflicts of interest.

## AUTHOR CONTRIBUTIONS

LCC performed immunofluorescence; TEF and MG performed electron microscopy data acquisition; TCG performed manual segmentation of the tomograms; BOP, JP and MG designed the research study; LCC, MEH, JP, BOP and MG analysed the data and drafted the manuscript.
