# Supplementary figures and images for: Age‐related ultrastructural changes of the basement membrane in the mouse blood‐brain barrier
Source: J Cell Mol Med. 2018 Nov 19;23(2):819–27. doi: 10.1111/jcmm.13980 (PMC6349169; doi:10.1111/jcmm.13980)

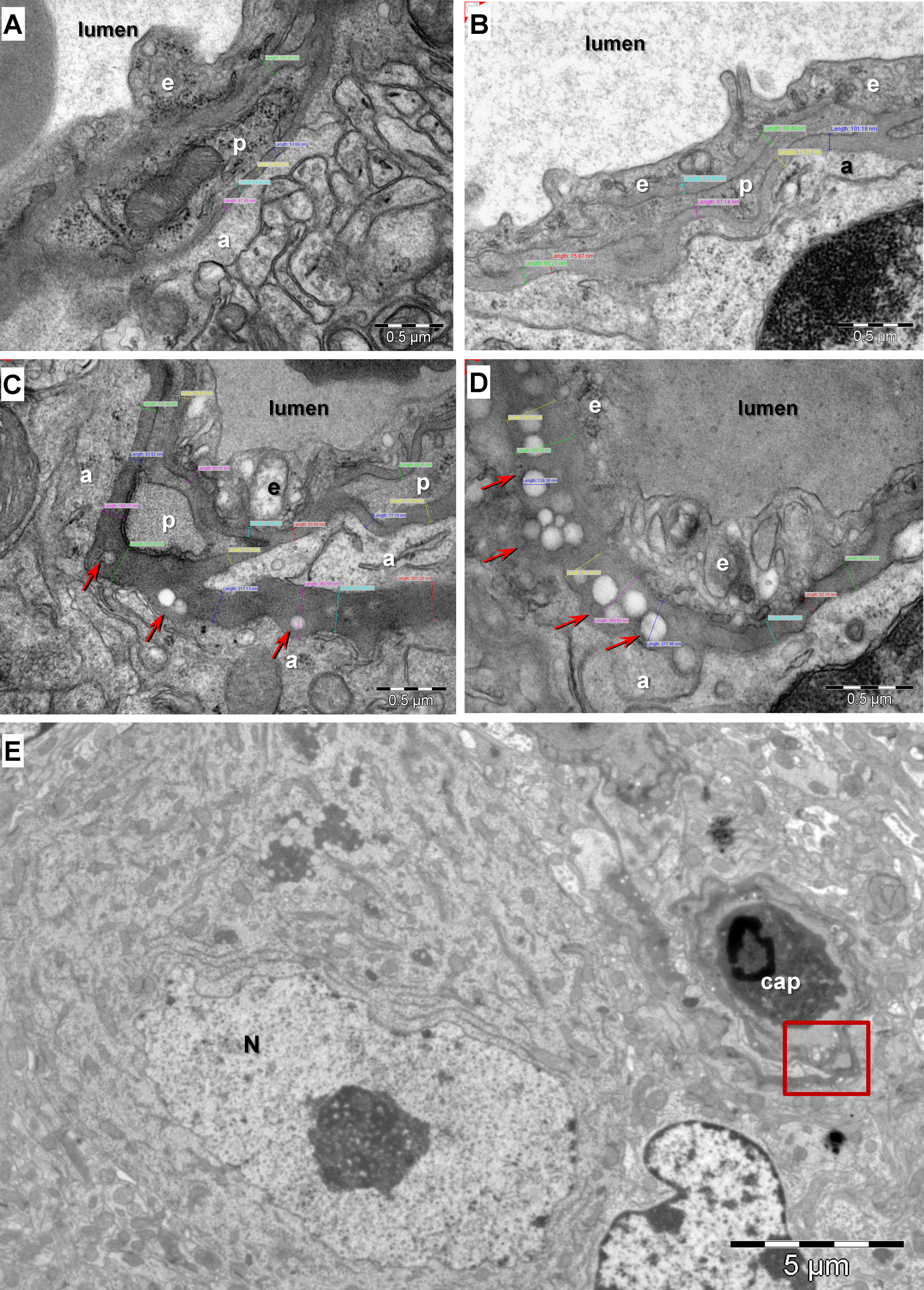

Supplement: Supplementary file 1 [file JCMM-23-819-s001.tif]

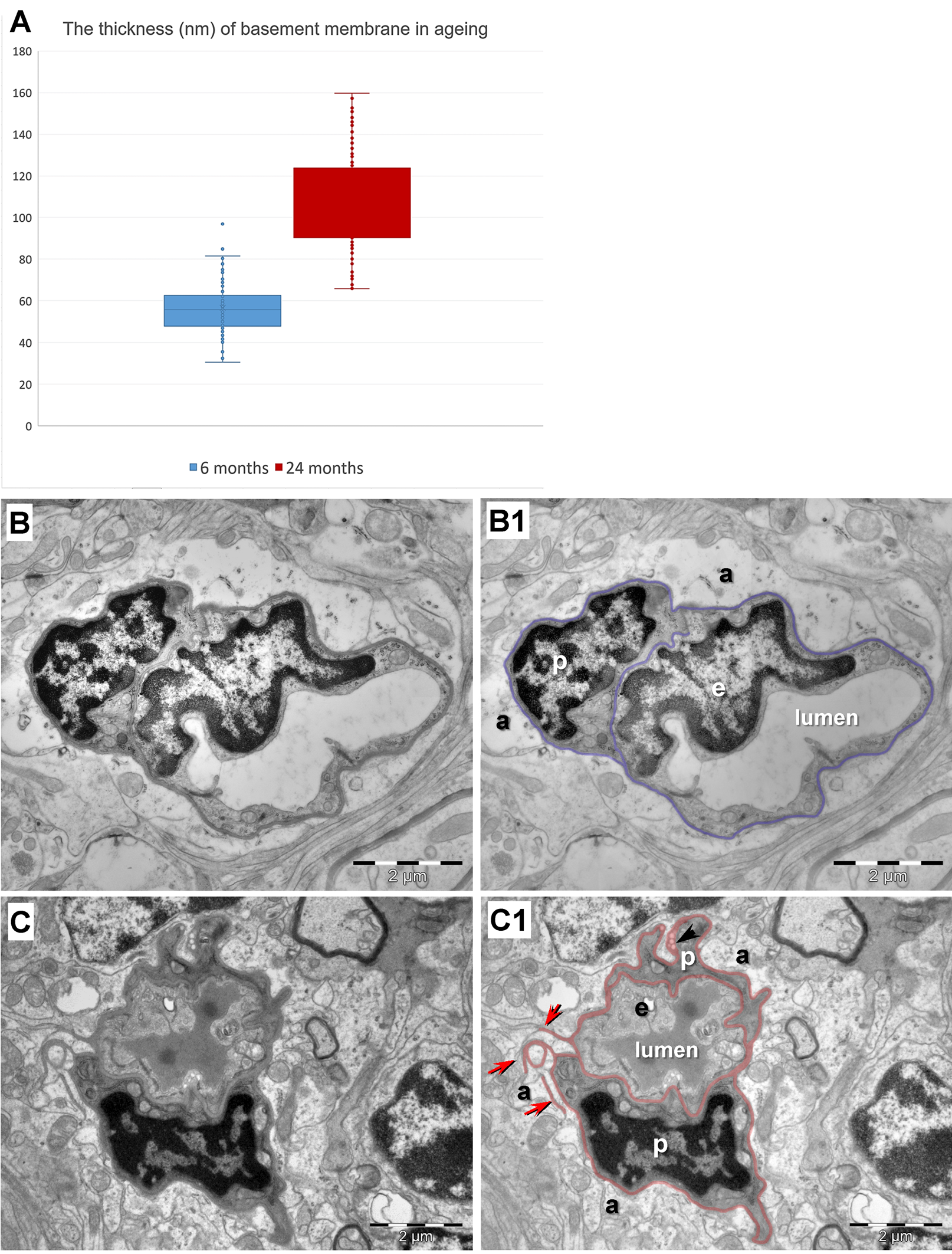

Supplement: Supplementary file 2 [file JCMM-23-819-s002.tif]

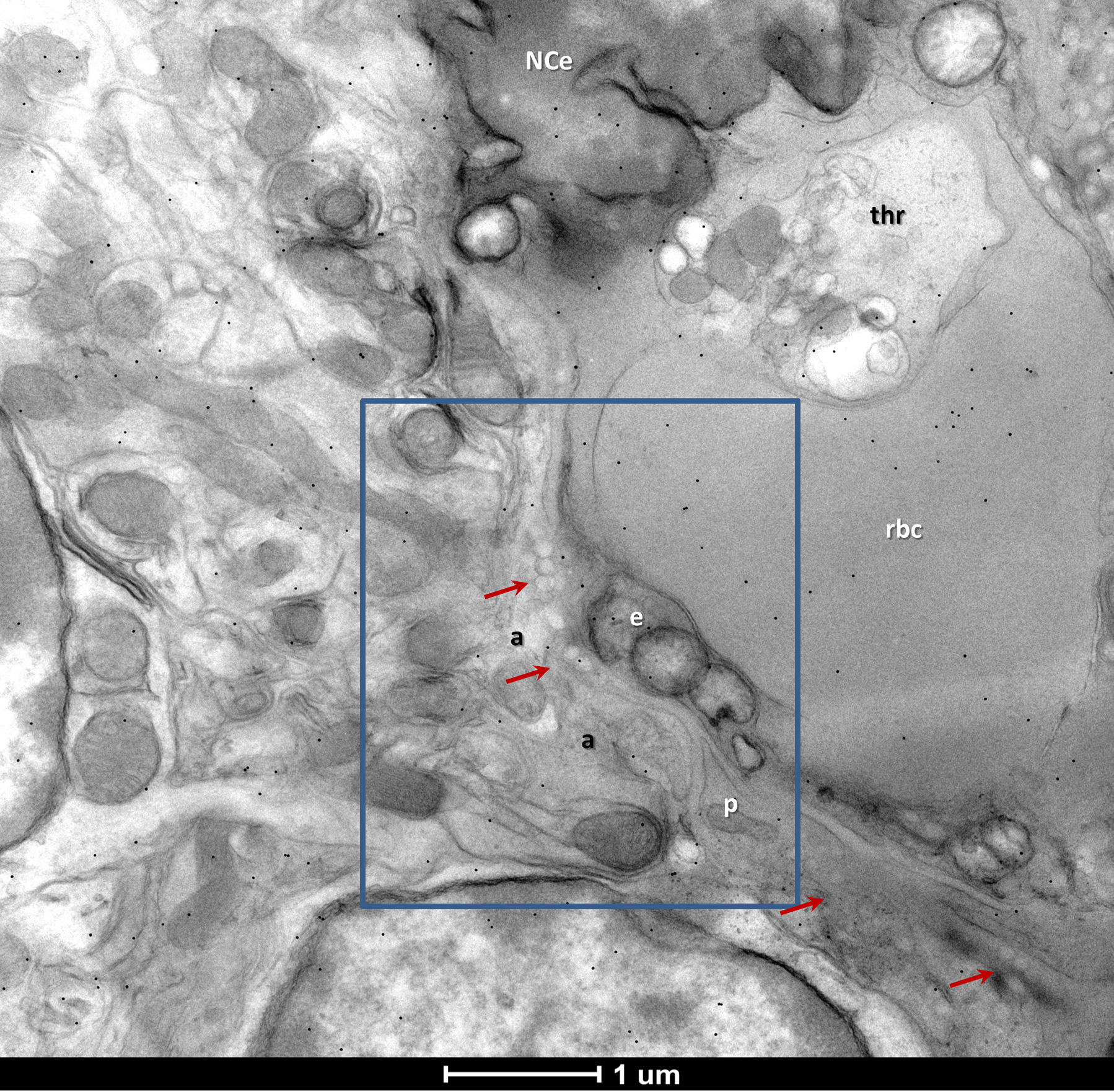

Supplement: Supplementary file 3 [file JCMM-23-819-s003.tif]

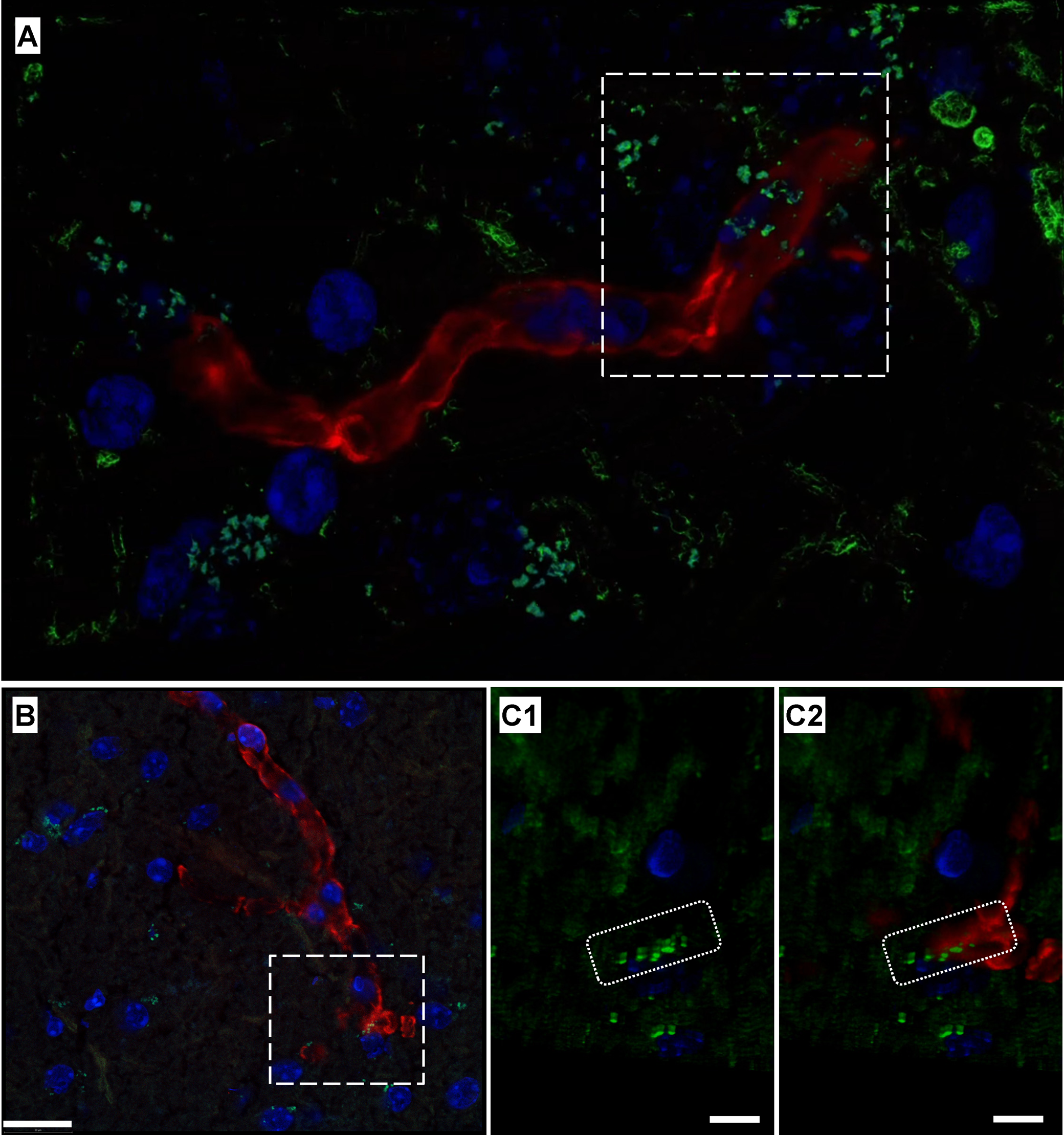

Supplement: Supplementary file 4 [file JCMM-23-819-s004.tif]
